# Comparison of 3,000 and 5,000 IU aXa/day certoparin in the prevention of deep-vein thrombosis after total hip replacement

**DOI:** 10.1186/1477-9560-10-10

**Published:** 2012-06-19

**Authors:** Peter Bramlage, Hans-Christoph Michaelis, Nima Melzer

**Affiliations:** 1 Institut für Pharmakologie und präventive Medizin, Menzelstrasse 21, Mahlow, 15831, Germany; 2 Novartis Pharma GmbH, Nürnberg, Germany

## Abstract

**Background:**

The aim was to investigate, whether 5,000 IUaXa/day certoparin lowers the incidence of deep vein thrombosis (DVT) in patients undergoing elective hip replacement surgery *vs.* 3,000 IUaXa/day. Double-blind, multicenter, randomised trial in 500 patients. Primary endpoint: incidence of symptomatic or asymptomatic DVT (bilateral ascending venography).

**Results:**

Mean age was 71 ± 10 years with a higher prevalence of previous DVT (8vs.4%) and pulmonary embolism (PE) (4vs.1%) in the high dose group. Mean duration of surgery was 82 ± 32 and 85 ± 36 min. DVT was detected in 28 (11.1%) of the low dose and 35 (14.1%) of the high dose group (p = n.s.). Combined distal-proximal DVT was observed in 5 (2%) and 4 (1.6%) patients respectively. No difference in bleeding events was found.

**Conclusion:**

This trial confirms prior data showing that the conventional dosage of 3,000 IU aXa is effective and safe for the prevention of venous thromboembolic events after hip replacement surgery.

## Background

Venous thromboembolism (VTE) is, without appropriate prophylaxis, a frequent complication in patients undergoing hip replacement with an incidence of 60% [1,2]. Even with pharmacological and physical prophylaxis there is a rate of 10-20% for asymptomatic distal, 5-10% for asymptomatic proximal, 2–5% of symptomatic thrombosis and of 0.3% for pulmonary embolism [3].

Against this background patients undergoing hip replacement surgery are a high risk group in itself and further stratification according to age, co-morbidity and other risk factors is not mandated. To cope with this risk current guidelines on the prevention of venous thromboembolism recommend to prefer low molecular weight heparin (LMWH) or Fondaparinux over unfractionated heparin (UFH) for peri- and postoperative prophylaxis [3]. Since the various LMWHs differ in regard to molecular weight and pharmacological properties different treatment regimes and doses may be recommended. Certoparin is one of these LMWHs that is currently registered for preventing VTE in high risk patients (including orthopaedic surgery), patients at medium risk (including general surgery), and after ischemic insult, as well as medical patients with acute illness of various origin at a fixed body-weight independent dose of 3,000 IE aXa/d. Certoparin was shown to be efficacious and safe in surgical and non-surgical patients [4-9]. According to a meta-analysis on the dosing of various other LMWH it was however shown, that there is a strong correlation between LMWH dosage and the risk of DVT (p = 0.03) [10].

In a prior randomized, controlled trial by Adolf *et al.*[8] in 341 patients undergoing hip replacement, the rate of deep vein thrombosis, detected by bilateral ascending venography, was not different in the 3,000 IE aXa/d (n = 17, 9.9%) and the 5,000 IE aXa/d group (n = 16, 9.5%). Bleeding complications were also not significantly different between groups, except for cell saver volumes (p < 0.001). We aimed to confirm these data in a further randomized, controlled trial, comparing the efficacy and safety of standard certoparin prophylaxis (3,000 UI aXa/d) to high dose certoparin prophylaxis (5,000 IU aXa/d) in patients with elective hip replacement.

## Results

### Patient characteristics

A total number of 500 patients were enrolled into the trial. 252 patients were allocated to 3,000 IU aXa/d Certoparin (low dose group) and 248 to 5,000 IU aXa/d Certoparin (high dose group). Baseline characteristics of the patients are summarized in Table 1. They had a mean age of 70.6 ± 9.6 (low dose) and 71.7 ± 10.6 (high dose) years. Approximately one third of the patients were male (36.9% *vs.* 33.1%; respectively). Most frequent predisposing risk factors in both groups were varices (35.7% *vs.* 35.1%), leg edema (27.0% *vs.* 35.1%) and previous hip surgery (30.2% *vs.* 33.9%). No relevant differences were found between the two groups except in the previous rate of DVT (p = 0.047) and previous pulmonary embolism (p = 0.011) with significantly higher incidence rates in the high dose certoparin group.

**Table 1:** Patients characteristics

	**3,000 IU aXa** **(n = 252)**	**5,000 IU aXa** **(n = 248)**	**p-value**
Age (years) mean ± SD	70.6 ± 9.6	71.7 ± 10.6	n.s.
Sex (male)	93 (37%)	82 (33%)	n.s
Weight (kg) mean ± SD	71.3 ± 15.3	71.4 ± 14.9	n.s
Predisposing risk factor			
Smoker	43 (17%)	41 (17%)	n.s.
Previous hip surgery	76	84	n.s.
Previous DVT	9 (4%)	19 (8%)	0.047
Previous pulmonary embolism	2 (1%)	11 (4%)	0.011
Obesity	46 (19%)	58 (23%)	n.s.
Hypertension	57 (23%)	71 (29%)	n.s.
Frequent bleeding episodes	2 (0.8%)	0 (0.0%)	n.s.
Leg edema	68	87	n.s.
Varices	90 (36%)	87 (35%)	n.s.
Varicous ulcer	12 (4.8%)	18 (7.3%)	n.s.

DVT, deep venous thrombosis; SD, standard deviation: n.s., not significants.

### Surgical intervention

Cemented hip prosthesis was the most predominant type of hip replacement (212 *vs.* 198 for Certoparin 3,000 IU aXa/d and Certoparin IU 5,000 aXa/d; respectively), followed by non-cemented hip replacement (32 *vs.* 28) (Table 2). The mean duration of the surgery was 81.9 ± 31.5 min in the low-dose group and 84.5 ± 36.0 min in the high dose group. No relevant differences were found between groups concerning duration and type of surgery and anaesthesia techniques.

**Table 2:** Surgical intervention and anaesthesia

	**3,000 IU aXa/d** **Certoparin (n = 247)**	**5,000 IU aXa/d** **Certoparin (n = 232)**
**Surgical intervention**		
Duration (min)	81.9 ± 31.5	84.5 ± 36.0
Type*		
Cemented	212 (86%)	198 (85%)
Not-cemented	32 (13%)	28 (12%)
Hybrid (cem. stem, not-cem cup)	1 (0.4%)	4 (2%)
Others	2 (0.8%)	2 (0.8%)
**Type of anaesthesia**		
Duration (min	99.7 ± 34.3	102.8 ± 41.1
General anaesthesia	143 (57.9%)	129 (55.6%)
Epidural/spinal anaesthesia	40 (16%)	37 (15.9)
General + epidural/spinal anaesth.	58 (24%)	71 (7%)

*Values refer to number of patients; n.a., not available.

### Incidence of thromboembolic events

**Figure 1:**
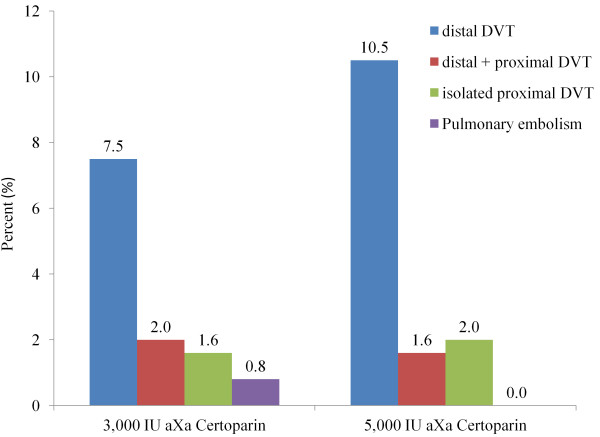
**Incidence of thromboembolic events *: DVT, deep venous thrombosis.* **

The incidence rates of thromboembolic events are shown in Figure 1. Fifty-nine patients in the low-dose group and 43 in the high-dose group refused venography, did not receive venography for medical reasons or were withdrawn from the study for other reasons. Deep vein thrombosis (DVT) was diagnosed by venography in 28 (11.1%) patients receiving low dose and in 35 (14.1%) patients receiving high dose certoparin. Combined distal-proximal DVT was observed in five patients (2.0%) receiving 3,000 IU aXa/d and in four (1.6%) receiving 5,000 IU aXa/d. Differences between dosing groups were statistically non-significant. There were two cases of suspected pulmonary embolism in the low dose and none in the high dose group (p = 0.16), which were based on a clinical diagnosis only.

### Bleeding complications

Peri-operative bleedings occurred in 105 patients of the low dose group and in 120 patients of the high dose group. Significant more bleedings considered as heparin-related were observed in the high dose group compared to the low dose group (p = 0.01; Table 3). In contrast the low dose group required significantly more transfusions, packed cells, fresh frozen plasmas and had higher blood losses (p < 0.001). No differences between the groups were found regarding postoperative bleeding parameters (Table 3).

**Table 3:** Bleeding Parameter

	**3,000 IU aXa/d** **Certoparin (n = 247)**	**5,000 IU aXa/d** **Certoparin (n = 232)**	**p-value**
**Intraoperative bleeding parameters**			
Bleeding events	105	120	n.s
Bleedings considered heparin-related	24	43	0.01
Transfusions	98	118	n.s.
whole blood (ml; mean ± SD)	885 ± 297	1033 ± 505	<0.001
Packed cells (ml; mean ± SD)	700 ± 392	504 ± 253	<0.001
Fresh frozen plasma (ml; mean ± SD)	617 ± 165	480 ± 234	<0.001
Drain loss (ml; mean ± SD)	333 ± 241	361 ± 266	n.s.
Other blood loss (ml; mean ± SD)	467 ± 250	225 ± 75	<0.001
Wound hematoma	4	1	n.s.
Wound oozing	130	128	n.s.
Wound revision	0	1	n.s.
Postoperative bleeding parameters*			
Bleeding events	170	167	n.s.
Bleeding related discontinuation of prophylaxis	12	15	n.s.
Bleedings considered heparin-related	103	119	n.s.
Transfusions	95	101	n.s.
Evacuation of wound hematoma	7	4	n.s.
Other reoperations	5	4	n.s.

*Values refer to number of patients.

### Laboratory parameters

aPTT, HEPTEST and platelet counts were performed pre-operatively and post-operatively at day 1, 5 and last postoperative day. No significant aPPT prolongation was found for either low or high dose group, no significant difference in the number of platelet drop downs <100.000/μl was detected. HEPTEST values at day 5 were 0.21 ± 0.12 IU/ml under 3,000 IU and 0.28 ± 0.18 under 5,000 IU Certoparin (p < 0.001).

The results for the coagulation parameters aPTT, HEPTEST and platelets are shown in Table 4**.** aPTT values of both groups were not significantly different, whereas HEPTEST values were significantly higher under 5,000 IU aXa/d. Platelet count beyond 100,000/μl was more often in the high-dose group, but without being statistically significant.

**Table 4:** Laboratory Parameter

	**3,000 IU aXa/d** **Certoparin (n = 247)**	**5,000 IU aXa/d** **Certoparin (n = 232)**	**p-value**
**aPPT (sec; mean ± SD)**			
Preoperatively	42.5 ± 13.1	42.3 ± 12.0	n.s.
Post-op day 1	49.2 ± 17.1	51.9 ± 18.5	n.s.
Post-op day 5	43.9 ± 10.9	45.5 ± 14.3	n.s.
Last post-op day	44.7 ± 19.6	45.0 ± 19.7	n.s.
**HEPTEST (IU/ml; mean ± SD)**			
Preoperatively	0.16 ± 0.18	0.21 ± 0.29	0.021
Post-op day 1	0.27 ± 0.16	0.33 ± 0.17	<0.001
Post-op day 5	0.21 ± 0.12	0.28 ± 0.18	<0.001
Last post-op day	0.21 ± 0.12	0.29 ± 0.18	<0.001
**Number of platelet counts < 100,000/μl**			
Preoperatively	0	1	n.s.
Post-op day 1	1	6	n.s.
Post-op day 5	3	0	n.s.
Last post-op day	1	1	n.s.

*Values refer to number of patients; ** (ml; mean ± SD).

### Adverse events

During the course of the study two patients of each group died. In the low-dose group one patient died due to massive gastrointestinal bleeding and another due to myocardial infarction. In the high dose group two patients died due to coronary insufficiency resp. myocardial infarction. Table 5 summarizes the serious adverse events. Differences between treatment groups were non-significant.

**Table 5:** Incidence of SAE’s according to EC-GCP-guidelines

**Serious Adverse Events**	**3,000 IU aXa/d** **Certoparin**	**5,000 IU aXa/d** **Certoparin**
**Death**	2	2
**Bleeding-related SAE**		
Wound hematoma	2	5
Wound hematoma infection	-	-
Wound infection	-	-
Bleeding events	3	9
**Non-Bleeding-related SAE**		
Chest pain	1	1
Myocardial infarction	1	2
AV-Block	0	1
Pulmonary embolism	0	1
Staphylococcus aureus infection	3	0
Metastatic carcinoma	-	-
Coxarthrosis	-	-
Prostata-adenoma	-	-
Retention of urine	-	-
Implant dislocation	2	3
Implant change	0	1
Second hip surgery	3	0
Further fracture	0	1
Any other re-operation	0	1

SAE, Serious Adverse Event; all differences are statistically not significant.

## Discussion

The present study confirms prior randomized, controlled trial data that 5,000 IU aXa/d certoparin are no more effective than 3,000 IU aXa/d in preventing thromboembolic events in patients undergoing elective hip replacement. It also confirms that bleeding complications are nominally increased in the high-dose group. Therefore current dosing patterns recommending 3,000 IU aXa/d for the prevention of venous thromboembolism in patients with total hip replacement are reinforced.

### VTE prevention and bleeding complications

For patients undergoing elective total hip replacement (THR), the recent ACCP guidelines recommend the routine use of one of the following anticoagulant options [11]: LMWH, fondaparinux, apixaban, dabigatran, rivaroxaban, low-dose unfractionated heparin (LDUH), adjusted-dose VKA, aspirin (all Grade 1B), or an intermittent pneumatic compression device (IPCD) (Grade 1 C). The recommendation for LMWH is based on a number of large trials [12-16] that have reported all DVT rates of about 13.7%, proximal DVT of 3.4% and pooloed rates of major bleeding in 5.3% of patients.

The present DVT rates are in agreement with the aforementioned rates reported for LMWH (13.7%) and confirm prior results published by Adolf et al. in 342 patients with elective hip replacement [8]. In the Adolf trial [8], comparing also standard (3,000 IU aXa/d) and high dose (5,000 IU aXa/d) certoparin, the rates of DVT were nominally lower (standard dose: 9.9% *vs.* high dose: 9.5%) than in our study (11.1% *vs.* 14.1%, respectively). The higher incidence of DVT in the high-dose certoparin group in our study may be explained by the significantly higher portion of patients with previous DVT and PE (Table 1). Incidence rates were also comparable to another certoparin study comparing 3,000 IU aXa/d as a fixed-dose combination with dihydroergotamin 0.5 mg (DHE), evaluated against dose adjusted unfractionated heparin (UFH) subcutaneously every 8 hours. DVT was detected in 12.0% in the LMWH/DHE group, no pulmonary embolism was detected [17].

Certoparin at a dose of 3,000 aXa/d IU is registered for VTE prophylaxis in surgical and non-surgical patients. This registration is based on trials comparing different doses and dosing regimens between 3,000, 5,000 IU certoparin and other comparators [4,5,8,17]. They all demonstrated non-inferiority of 3,000 IU aXa/d certoparin *versus* the chosen comparator. Repetitive administrations of 3,000 IU antiXa certoparin after 12 or 24 hours led to no cumulation.

The study also confirms that rates of bleeding complications are similar for both dosing groups although heparin-related bleeding was more frequent in the high-dose group. This also fits with the data provided by Adolf *et al*[8]. We further confirmed the observation of the trial by Adolf et al., that in the high dose group more whole blood transfusions were given but less cell saver and FFP volumes compared to the low dose group. Results are difficult to compare however to rates of major bleedings reported for LMWH (5.3%) because of different definitions used.

### Limitations

The present data have been acquired in the late 1990’s and clinical treatment pathways and techniques for (elective) hip replacement have changed since then. This is a major limitation of the present trial which was planned and conducted at times when the composite endpoint of VTE related death, deep venous thrombosis and pulmonary embolism was not yet defined as the generally accepted primary endpoint of such studies. The same limitation has to be considered for the safety endpoint, which nowadays includes predefined major and minor bleeding events, that can´t be easily compared to the present bleeding criteria, which are mentioned in Table 3. Further, while the main study outcome was assessed by an independent radiologist, unaware of the dosis assignment no independent adjudication committee was employed. We are convinced however that the current data should be published, to make them accessible to the scientific community for transparency purposes.

## Conclusions

This clinical trial confirms available data on maximal antithrombotic efficacy and an excellent safety profile of 3,000 *versus* 5,000 IU aXa/d certoparin in the prevention of thromboembolic events after elective hip replacement. The data are in line with the incidence rates of VTE complications after major orthopaedic surgery when low molecular weight heparins are used for prophylaxis.

## Methods

This study was a double-blind, multicenter, prospective, randomised trial. It was approved by an independent ethics committee and performed according to the Declaration of Helsinki. Prior to enrolment patients` written informed consent was obtained.

### Patients

Patients aged at least 40 years and of either gender scheduled for elective primary hip replacement were eligible for inclusion. Exclusion criteria were severe renal impairment (serum creatinine > 200 μmol/l) or hepatic dysfunction (INR > 1.2), severe hypertension (BP > 200/105 mmHg), known bleeding diathesis, allergy to heparin or its fragments, allergy to iodine containing radio-opaque contrast media, hyperthyroidism, premenopausal women being pregnant or not actively preventing pregnancy, breast feeding and participation in a clinical trial four weeks prior to inclusion in this trial as well as usage of drugs which might influence coagulation within seven days prior to surgery.

### Treatment allocation

After inclusion patients were randomly allocated to one of two treatment groups receiving subcutaneously either 3,000 or 5,000 IU aXa of certoparin once a day. The injections were applied in the morning at least two hours prior to surgery. The prophylactic treatment was continued for a minimum of 8 up to a maximum 16 days.

### Endpoint definition

The efficacy endpoint was the incidence of asymptomatic or symptomatic proximal or distal DVT. Bilateral venography was performed between the 8^th^ and the 16^th^ postoperative day in all asymptomatic patients. In patients with clinical signs suggesting DVT, bilateral ascending venography was performed immediately after the symptoms were recognised (early venography). Venographies were blinded and examined by an independent expert radiologist according to clinical routine standard procedures. Clinical signs of pulmonary embolism (PE) were to be verified by pulmonary scintigraphy.

### Bleeding events and other safety parameters

For safety, adverse events especially bleeding complications were observed. Intraoperative blood loss was assessed by the surgeons. In addition the blood loss from drains in the whole postoperative prophylaxis period was documented. Surgical wounds were examined daily for hematomas and wound oozing. All intra- and postoperative transfusions, bleeding related discontinuations and reoperations as well as all serious or non-serious adverse events, observed during the prophylactic treatment, were documented. HEPTEST, aPTT, and thrombocyte count were determined preoperatively, on the first and the fifth postoperative day as well as on the day of discharge. There was no screening for thrombocyte directed antibodies however.

### Statistics

The sample size calculation was made on the basis of available literature at the time of study conception. VTE incidence after hip surgery was estimated occurring despite prophylaxis in about 30%. An assumed decrease of that rate of about 10% was estimated for the high dose group. A sample size of 500 patients was calculated including planned drop outs to achieve a power of 90% at a significance level of 0.05. The incidence of thromboembolic events were calculated on the basis of an intent-to-treat analysis. Statistical testing was performed with the unpaired *t*-test or with the uncorrected Chi-square test.

## Competing interests

P. Bramlage reports to have received compensation for consulting and lecture honoraria from Novartis. H.C. Michaelis and N. Melzer are employees of the sponsor.

## Authors' contributions

HCM was involved in the planning and conduct of the study. NM and PB drafted the manuscript and requested additional analyses. All authors revised the manuscript for important intellectual content and approved the final version of the manuscript.
